# The Effect of Anesthetic Depth on the Occurrence of Emergence Delirium in Children Undergoing Strabismus Surgery: A Prospective Observational Study

**DOI:** 10.3390/biomedicines13010063

**Published:** 2024-12-30

**Authors:** Yea-Ji Lee, Jung-Won Hwang, Sang-Hwan Do, Hyo-Seok Na

**Affiliations:** 1 Department of Anesthesiology and Pain Medicine, Konkuk University Medical Centre, Seoul 05030, Republic of Korea; ladydaisy82@naver.com; 2 Department of Anesthesiology and Pain Medicine, Seoul National University Bundang Hospital, Seongnam 13620, Republic of Korea; jungwon@snubh.org (J.-W.H.); 00782@snubh.org (S.-H.D.)

**Keywords:** anesthesia, general, bispectral index, child, emergence delirium, surgical procedure, ophthalmic, strabismus

## Abstract

Background/Objectives: Emergence delirium (ED) is one of the most frequent postoperative complications in pediatric patients after general anesthesia. In adults, a deeper intraoperative level of anesthesia has been reported as an independent predictor of postoperative delirium. However, the effect of anesthetic depth on ED has rarely been demonstrated in the pediatric population. We evaluated whether the depth of general anesthesia could affect the occurrence of emergence delirium (ED). Methods: Patients aged 3–5 years, scheduled for strabismus surgery were enrolled in this prospective observational study. Intraoperative bispectral index (BIS) was monitored, and the pediatric anesthesia emergence delirium (PAED) scale was evaluated. When the PAED scale was 10 or more, it was designated as an ED case. Results: According to the intraoperative mean BIS range, enrolled patients were divided into two groups: the low BIS (BIS < 40; *n* = 28) and the normal BIS (BIS 40–60; *n* = 34) group. The incidence of ED was comparable between the two groups (67.6% vs. 67.9%, odds ratio = 0.99, 95% CI = 0.34–2.89, *p* = 0.986). Conclusions: The intraoperative anesthetic depth did not seem to affect the occurrence of ED in pediatric patients undergoing strabismus surgery under general anesthesia. Future studies with a larger sample size are necessary for more authentic results.

## 1. Introduction

Cautious and precise anesthetic care is required for pediatric patients throughout the intraoperative and post-anesthetic recovery periods. Emergence delirium (ED) is one of the postoperative problems, along with postoperative pain, hypothermia, bleeding, and so on [1]. ED is commonly observed in preschool-aged children, and its incidence ranges from 10 to 80% [2,3]. It is characterized by a dissociated state of consciousness, in which children are inconsolable, irritable, uncooperative, thrashing, crying, moaning, or incoherent [1,4].

Although the exact etiology of ED remains unclear, several predisposing factors include rapid emergence from general anesthesia, postoperative pain, use of volatile anesthetics, head and neck surgeries, agitation on induction, airway obstruction, and hyperthermia or hypothermia [5,6,7].

Treatment and prevention of ED are clinically important because ED is strongly associated with postoperative delirium (POD), which leads to prolonged hospital stays and increased morbidity, mortality, and the need for institutionalization of adult patients [8,9]. To predict ED, the ED risk scale was developed for children receiving sevoflurane anesthesia [10]. Identified biomarkers collected via blood samples and monitoring electroencephalogram (EEG) patterns during surgery would also be helpful to predict ED [11,12]. Several scale tools for assessing ED in children and adults have been proposed. Among them, the pediatric anesthesia emergence delirium (PAED) scale, developed in 2004, is the most commonly used in pediatric ED studies [13].

Several studies have reported that anesthetic depth is associated with the occurrence of POD. There are two main techniques for monitoring the depth of anesthesia: (1) processed EEG, such as bispectral index (BIS), entropy, and patient state index, and (2) middle latency auditory evoked potential (AEP) [14]. In adults, a deeper intraoperative level of anesthesia has been reported as an independent predictor of POD [15]. Early postoperative cognitive dysfunction (POCD) declined in patients with properly controlled anesthetic depth using AEP [16]. BIS is the most investigated technique that processes the EEG data collected through a sensor placed on the patient’s forehead [14,17]. The collected EEG information is transformed into values between 0 and 100. Each BIS range represents a specific level of consciousness: (1) awake (80–100), (2) light to moderate sedation (60–80), (3) general anesthesia (40–60), (4) deep hypnotic state (20–40), and (5) <20: burst suppression. BIS value ‘0’ means flat-line EEG [18]. A few studies have demonstrated that BIS-guided anesthesia resulted in less anesthetic exposure, which could lead to lighter anesthesia [19,20]. A recent study showed that deeper (BIS target 35) anesthesia was associated with a higher incidence of POD than lighter (BIS target 50) anesthesia. However, the effect of anesthetic depth on ED has rarely been evaluated in the pediatric population [21].

We assumed that the requirement for anesthetic agents and the consequent intraoperative depth of anesthesia would differ from patient to patient. As a result, the discrepancy in the intraoperative depth of anesthesia might affect the ED incidence. Thus, in this study, we observed the requirement for anesthetic agents and the intraoperative anesthetic depth in pediatric patients undergoing strabismus surgery and evaluated whether the occurrence of ED was different according to the intraoperative anesthetic depth.

## 2. Materials and Methods

This prospective observational study was conducted in accordance with the Declaration of Helsinki, and the protocol was approved by the local Institutional Review Board (IRB No. B-1503-289-004). The study protocol was registered in ClinicalTrials.gov (NCT02521259).

### 2.1. Study Subjects

Patients aged 3–5 years, scheduled for strabismus surgery, were recruited for this study. The exclusion criteria were as follows: American Society of Anesthesiologists physical status classification (Table 1) [22] of greater than III, current abnormal cognitive status, mental retardation, previous neurosurgical history, and any co-existing diseases that could affect the mental status of patients. We obtained written informed consent from the participants’ parents prior to study initiation.

### 2.2. Procedures

No premedication for anxiolysis was administered. In the pre-anesthetic holding area, patients and their parents were briefed on the process of volatile induction and maintenance anesthesia (VIMA). The intravenous line was not placed preoperatively, and patients were accompanied by one of their parents when entering the operating room. Upon arrival at the operating room, pulse oximetry was applied to the index finger. The children were encouraged to breathe voluntarily through a facial mask by a parent for a few minutes. Initially, oxygen and nitrous oxide (inspired oxygen fraction = 0.4, total flow rate = 6 L/min) were supplied through a facial mask, and the inspired sevoflurane concentration gradually increased. After loss of consciousness, manual mask ventilation with oxygen and 8 vol% of sevoflurane was performed by an anesthesiologist. Concurrently, monitoring of non-invasive arterial pressure, electrocardiogram, and BIS were started. During manual mask ventilation, the intravenous line was placed on the upper or lower extremity of the patients, and a single bolus of 10 μg/kg of alfentanil was given. No neuromuscular blocking agent was used. According to the body weight, a laryngeal mask airway (LMA) Flexible with size #2 or #2.5 (Teleflex Medical, Co. Westmeath, Ireland) was inserted to secure the airway, and anesthesia was maintained using sevoflurane with an oxygen and nitrous oxide mixture (inspired oxygen fraction = 0.4, total flow rate = 3 L/min).

One independent anesthesiologist, who did not participate in the anesthetic care, only monitored and assessed the intraoperative BIS value, burst suppression, and suppression ratio (the percentage of EEG suppression) during the main surgical procedure (incision-surgical dressing). If burst suppression was observed during this period, the study was suspended, and the patient dropped out. We set the BIS smoothing rate (the period used to calculate the BIS value by averaging artifact-free data) to 15 s mode. The other anesthesiologist, who was blinded to the intraoperative BIS, provided routine anesthetic care to patients and controlled the sevoflurane concentration for maintaining hemodynamic stability. If intraoperative blood pressure changed by 20% from the patients’ baseline value, the concentration of sevoflurane was adjusted. The inhalation concentration of sevoflurane was recorded every 5 min. For each patient, we plotted a graph with anesthesia time and inhalation concentration of sevoflurane on the X- and Y-axes, respectively. Using integral calculus, we calculated the area under the curve for sevoflurane concentration, which was divided by the total anesthesia time (min). This concentration was regarded as the mean concentration of inhaled sevoflurane during anesthesia [23]. When bradycardia (heart rate < 40 beats per minute) was observed intraoperatively, atropine was administered. At the end of the operation, we gave all patients a single bolus of 0.5 mg/kg of ketorolac with the maximum dose not exceeding 30 mg. After finishing the operation, sevoflurane was discontinued, and patients’ lungs were ventilated with 100% oxygen until spontaneous ventilation was restored. After patients showed voluntary movement, spontaneous ventilation with tidal volume > 5 mL/kg, SpO_2_ > 95%, and blood pressure and heart rate within 20% of their baseline values were maintained for over a minute, the LMA Flexible was removed, and patients were transferred to the post-anesthetic care unit (PACU). To minimize inter-subject variability, every anesthetic management was performed by the same anesthesiologist.

### 2.3. Assessment of ED

In the PACU, investigators who were blinded to the intraoperative BIS and were not involved in anesthetic care assessed ED. After arriving at the PACU, patients’ ED was measured using the PAED scale (Table 2) [24]. To minimize the disadvantages of the PAED scale, including intrinsic subjectivity in assessment and suboptimal interrater reliability [25], investigators carried out several rehearsals before the trial began. When the total PAED score was 10 or greater, we regarded the patient as having ED and gave 0.5 μg/kg of fentanyl intravenously to subside ED. The PAED score was evaluated three times every 10 min (T1, on arrival at the PACU; T2, 10 min after T1; T3, 10 min after T2). The mean PAED score is the average value of PAED scores from T1, T2, and T3. The peak PAED score is the highest value among the PAED scores from T1, T2, and T3.

After the clinical trial was over, the intraoperative BIS value was disclosed, and the mean value was calculated. The patients were divided into two groups based on the intraoperative mean BIS value: the low BIS (<40) and the normal BIS (40–60) group.

### 2.4. Statistical Analysis and Sample Size Calculation

The primary outcome was the incidence of ED determined by the PAED score at the PACU. The secondary outcomes were the mean inhaled concentration of sevoflurane, the mean PAED score, and the proportion of patients who were treated with fentanyl twice or more for relieving ED at the PACU.

The maximum incidence of ED after pediatric ophthalmic surgery was observed to be 79% in the pilot study. For the sample size estimation, it was assumed that the incidence of ED would be 44% (result obtained from the pilot study) in the normal BIS group. Using the statistical method, 60 patients and a 10% attrition rate (8 patients) were calculated, resulting in a total of 68 patients for a power of 80% and a type 1 error of 5%.

The normal distribution of collected data was tested using the Shapiro–Wilk test. All data were expressed as the mean ± standard deviation (SD) or number (%). The correlation between the intraoperative BIS and the highest PAED score was examined by Pearson correlation analysis. The PAED scores between the groups were analyzed with repeated measures ANOVA; the Mann–Whitney U-test was performed at each time point if the results of repeated measures ANOVA showed a significant difference. The difference in the incidence of ED or rescue medication was compared using the Chi-square test. The relative risk (RR) and 95% confidence interval (CI) were calculated. SPSS software (ver. 21.0, IBM Co., Armonk, NY, USA) was used for statistical analyses, and a *p* value of less than 0.05 was considered statistically significant.

## 3. Results

### 3.1. Characteristics of the Sample

**Figure 1:**
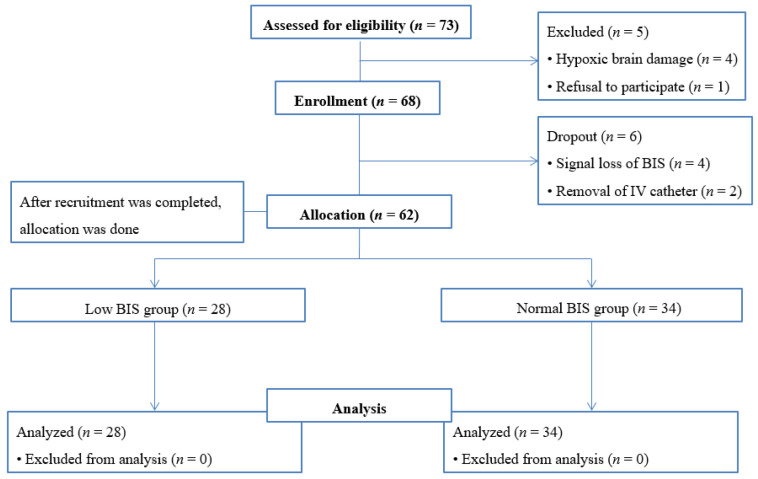
Flow diagram of patients’ enrollment.

Seventy-three patients were assessed for eligibility, and a total of 68 patients were enrolled. Six patients were excluded during the recruitment (4 due to intraoperative signaling defects of the BIS sensor, 2 due to accidental removal of the intravenous catheter during the recovery period). After the study protocol was completed, allocation was done according to the intraoperative mean BIS value. Sixty-two patients were allocated into two groups: 28 in the low BIS group and 34 in the normal BIS group, respectively (Figure 1).

The characteristics of the patients, surgery, and anesthesia are presented in Table 3. The mean BIS value was significantly lower in the low BIS group than in the normal BIS group (32.9 ± 4.9 vs. 51.0 ± 6.9, *p* < 0.001), and the mean concentration of inhaled sevoflurane was significantly higher in the low BIS group than in the normal BIS group (3.4 ± 0.7 vol% vs. 2.4 ± 0.7 vol%, *p* < 0.001).

### 3.2. PAED Score Analysis in PACU

The incidence of ED was 67.6% in the low BIS group and 67.9% in the normal BIS group (RR = 0.99, 95% CI = 0.34–2.89, *p* = 0.986). The result showed no statistical significance.

**Figure 2:**
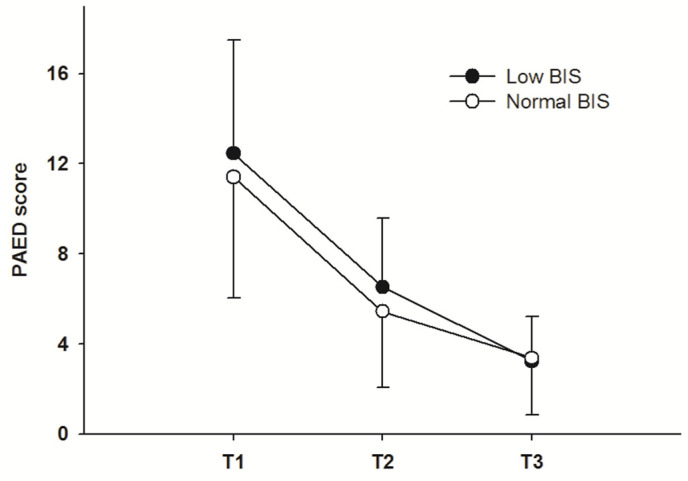
PAED score variations. Each white and black dot indicates the PAED score at each time point of measurement. T1, arrival at PACU; T2, 10 min after PACU arrival; T3, 20 min after PACU arrival. PACU, post-anesthetic care unit; PAED, pediatric anesthesia emergence delirium.

The PAED scores at each measurement point were similar between the two groups (Table 4). The correlation coefficient between the intraoperative mean BIS and the highest PAED score was −0.18 (*p* = 0.166). The difference in PAED scores between the two groups was not significant (*p* = 0.729). The change in PAED scores was not significantly different between the two groups (*p* = 0.648). The PAED scores of T2 and T3 decreased significantly compared to the PAED score of T1 in each group (*p* < 0.001) (Figure 2).

After the first fentanyl treatment, a smaller number of patients in the normal BIS group required an additional fentanyl treatment at the PACU compared to the low BIS group; however, the difference was not statistically significant (5.9% vs. 14.3%, *p* = 0.265).

## 4. Discussion

In this study, we found that the depth of intraoperative anesthesia has no effect on the incidence and severity of ED. We also discovered that ED in most cases resolved within 10 min after fentanyl treatment.

Strabismus surgery in children is known to present ED at a higher rate, about 40–86% [26,27,28]. In our study, when the cutoff value of PAED score for the presence of ED was set to 10 [24,29,30], the overall incidence was 67.8%. This finding was not so different from previously reported ones. When subgroups were classified according to the BIS level, the rate of ED was 67.6% in the low BIS group and 67.9% in the normal BIS group. Heather et al. reported similar results [31]. However, unlike our study, they deliberately controlled the intraoperative sevoflurane concentration to achieve the BIS level within an appropriate range. We adjusted the concentration of sevoflurane individually according to intraoperative vital signs, instead of using intentional control. A definite difference in BIS range between the groups was required to verify the effect of anesthetic depth on the occurrence of ED; however, intentionally increasing the depth of anesthesia seemed to be against medical ethics. Thus, an observational design was adopted, despite the mismatch of the allocated number of patients between the two groups.

Despite the nearly identical operational procedure among all patients, it was interesting to see that the requirement for sevoflurane was different from one individual to another. Kil et al. already reported that preoperative anxiety or pain sensitivity could influence the anesthetic requirement of each patient [32]. In the present study, the oculocardiac reflex seemed to be another possible cause for this inter-individual difference in anesthetic requirement. The inhaled concentration of sevoflurane might be increased in patients without an oculocardiac reflex for the purpose of stabilizing tachycardia or hypertension caused by surgical stimuli. On the other hand, the concentration of sevoflurane might not increase in patients who presented a bradycardic response, which was treated with atropine. Thus, the intraoperative requirement for sevoflurane was different among patients, resulting in different intraoperative BIS levels. However, the incidence of ED was comparable, which means that different anesthetic depth caused by different individual anesthetic requirements may not influence the occurrence of ED.

Burst suppression (BS) is an electroencephalographic pattern that shows alternating periods of high-voltage brain activity (bursts) and low-voltage brain activity (suppressions). BS is a state of no activity in the brain. Although deep anesthesia is known to be associated with POD, several studies reported that the average depth calculated with BIS values was not associated with POD, but the parameter of burst suppression was [33,34]. The occurrence of BS seems to be associated with an increased risk of POD [12,35,36]. Bruhn et al. demonstrated that suppression ratios (SR, the percentage of an EEG period with very low voltage) over 40% were linearly correlated with BIS values from 30 to 0 [37]. This result implies that the occurrence of BS would be increased with BIS values below 30. During the operation, we monitored SR to minimize the harmful effect induced by excessive anesthetics, and the observed ratio was nearly 0%. This low SR could affect the incidence of ED in our study. Further studies are necessary to demonstrate the effect of EEG patterns on ED.

Although ED was reported in patients receiving general anesthesia for painless evaluation, such as magnetic resonance imaging [38,39], postoperative pain still presents as an important risk factor for ED in children. Significant postoperative pain may result from strabismus surgery, which is one of the predisposing factors for ED. However, it is difficult to differentiate the irritability caused by postoperative pain from ED in pediatric patients. We gave alfentanil, nitrous oxide, and ketorolac as analgesic medications to the patients. Although controversy exists, there are reports describing the analgesic role of intraoperative nitrous oxide in acute postoperative pain [40,41]. Thus, the high PAED scores at the time of PACU arrival did not seem to be caused by insufficient surgical pain management.

Fentanyl has been established as an effective medication for the improvement of ED [42,43,44]. In most of our patients, ED was resolved with the single administration of fentanyl. It is worth noting that there were patients who still presented ED after the initial fentanyl administration. The proportion of patients who required a second fentanyl dose due to persistent ED was higher in the low BIS group. However, the enrolled population was too small to have statistical significance regarding this issue. It should be confirmed whether deeper intraoperative anesthetic depth may cause ED more severely and persistently in the next study.

Nevertheless, given that approximately 68% of patients received fentanyl to relieve ED at the PACU, prior administration of fentanyl before the presentation of ED would be an efficacious preventive strategy. The appropriate timing of the administration of fentanyl could be before discontinuation of anesthetics, during recovery from anesthesia, or immediately after extubation. There have been previously reported studies that successfully reduced the occurrence of ED by incorporating this preventive strategy—administration of fentanyl, dexmedetomidine, propofol, or ketamine prior to the presentation of ED [38,39,45,46]. However, close attention should be paid to respiratory depression or delayed recovery.

Our study has several limitations. First, the observational study design and the small sample size may limit the generalizability of our results and conclusions. Furthermore, equal distribution before enrollment cannot be expected because this study was designed as an observational study. For obtaining more reliable results, a further study should be carried out with a larger number of patients. Second, we did not measure the end-tidal sevoflurane concentration. When high concentrations of sevoflurane were administered for a long time, this could delay recovery from anesthesia. However, Oh et al. have already found that prolonged recovery did not influence the incidence of ED after sevoflurane anesthesia [47]. In addition, extubation was performed after confirming purposeful movement in the operating room. Thus, it is unlikely that there was a difference in consciousness between the two groups in the PACU. Lastly, although the assessment of ED was performed by pre-educated investigators to enhance interrater reliability, standardized practical tools are still needed for the objective evaluation of the severity of ED.

## 5. Conclusions

In conclusion, anesthetic requirement and intraoperative BIS were different among pediatric patients undergoing strabismus surgery. However, the depth of general anesthesia was not likely to affect the incidence of ED in children undergoing strabismus surgery. As expected, fentanyl was effective for relieving ED.

## Figures and Tables

**Table 1:** ASA physical status classification.

Classification	Definition
ASA I	A normal healthy patient
ASA II	A patient with mild systemic disease
ASA III	A patient with severe systemic disease
ASA IV	A patient with severe systemic disease that is a constant threat to life
ASA V	A moribund patient who is not expected to survive without the operation
ASA VI	A declared brain-dead patient whose organs are being removed for donor purposes

ASA, American Society of Anesthesiologists.

**Table 2:** Pediatric anesthesia emergence delirium (PAED) scale.

Item
1. The child makes eye contact with the caregiver 2. The child’s actions are purposeful 3. The child is aware of his/her surroundings 4. The child is restless 5. The child is inconsolable

Items 1, 2, and 3 are scored: 4 = not at all, 3 = just a little, 2 = quite a bit, 1 = very much, 0 = extremely. Items 4 and 5 are scored: 0 = not at all, 1 = just a little, 2 = quite a bit, 3 = very much, 4 = extremely.

**Table 3:** Characteristics of patients, surgery, and anesthesia.

	Low BIS (*n* = 28)	Normal BIS (*n* = 34)	*p* Value
Age (yr)	3.9 ± 0.79	4.1 ± 0.8	0.286
Gender (M/F)	13 (46.4)/15 (53.6)	16 (47.1)/18 (52.9)	0.961
Height (cm)	103.8 ± 9.3	104.9 ± 7.1	0.602
Weight (kg)	17.5 ± 3.1	17.8 ± 3.4	0.690
ASA (I/II)	27 (96.4)/1 (3.6)	32 (94.1)/2 (5.9)	0.673
Mean Sevo (vol%)	3.4 ± 0.7	2.4 ± 0.7	<0.001
Mean BIS	32.9 ± 4.9	51.0 ± 6.9	<0.001
Operation site (single/both)	12 (42.9)/16 (57.1)	16 (47.1)/18 (52.9)	1.000
Operation time (min)	20.2 ± 8.6	16.8 ± 5.8	0.066
Anesthesia time (min)	32.5 ± 11.2	30.2 ± 6.5	0.304

Data are expressed as mean ± SD or number (%). ASA, American Society of Anesthesiologists physical status classification; BIS, bispectral index; Sevo, inhaled sevoflurane concentration.

**Table 4:** PAED score.

	Low BIS (*n* = 28)	Normal BIS (*n* = 34)	*p* Value
T1	12.5 ± 5.0	11.4 ± 5.4	0.433
T2	6.5 ± 3.1	5.4 ± 3.4	0.203
T3	3.2 ± 2.0	3.4 ± 2.5	0.852

Data are expressed as mean ± SD or number (%). T1, arrival at PACU; T2, 10 min after PACU arrival; T3, 20 min after PACU arrival. PACU, post-anesthetic care unit; PAED, pediatric anesthesia emergence delirium.

## Data Availability

The data presented in this study are available on request from the corresponding author due to restrictions imposed by the Institutional Review Board which approved the study protocol.
